# Repeated Aerosolized-Boosting with Gamma-Irradiated *Mycobacterium bovis* BCG Confers Improved Pulmonary Protection against the Hypervirulent *Mycobacterium tuberculosis* Strain HN878 in Mice

**DOI:** 10.1371/journal.pone.0141577

**Published:** 2015-10-28

**Authors:** Seung Bin Cha, Woo Sik Kim, Jong-Seok Kim, Hongmin Kim, Kee Woong Kwon, Seung Jung Han, Seok-Yong Eum, Sang-Nae Cho, Sung Jae Shin

**Affiliations:** 1 Department of Microbiology, Institute for Immunology and Immunological Diseases, Yonsei University College of Medicine, Seoul, South Korea; 2 Brain Korea 21 PLUS Project for Medical Science, Yonsei University College of Medicine, Seoul, South Korea; 3 Division of Immunopathology and Cellular Immunology, International Tuberculosis Research Center, Changwon, South Korea; Colorado State University, UNITED STATES

## Abstract

*Mycobacterium bovis* bacillus Calmette-Guerin (BCG), the only licensed vaccine, shows limited protection efficacy against pulmonary tuberculosis (TB), particularly hypervirulent *Mycobacterium tuberculosis* (Mtb) strains, suggesting that a logistical and practical vaccination strategy is urgently required. Boosting the BCG-induced immunity may offer a potentially advantageous strategy for advancing TB vaccine development, instead of replacing BCG completely. Despite the improved protection of the airway immunization by using live BCG, the use of live BCG as an airway boosting agent may evoke safety concerns. Here, we analyzed the protective efficacy of γ-irradiated BCG as a BCG-prime boosting agent for airway immunization against a hypervirulent clinical strain challenge with *Mycobacterium tuberculosis* HN878 in a mouse TB model. After the aerosol challenge with the HN878 strain, the mice vaccinated with BCG via the parenteral route exhibited only mild and transient protection, whereas BCG vaccination followed by multiple aerosolized boosting with γ-irradiated BCG efficiently maintained long-lasting control of Mtb in terms of bacterial reduction and pathological findings. Further immunological investigation revealed that this approach resulted in a significant increase in the cellular responses in terms of a robust expansion of antigen (PPD and Ag85A)-specific CD4^+^ T cells concomitantly producing IFN-γ, TNF-α, and IL-2, as well as a high level of IFN-γ-producing recall response via both the local and systemic immune systems upon further boosting. Collectively, aerosolized boosting of γ-irradiated BCG is able to elicit strong Th1-biased immune responses and confer enhanced protection against a hypervirulent *Mycobacterium tuberculosis* HN878 infection in a boosting number-dependent manner.

## Introduction

Tuberculosis (TB), caused by *Mycobacterium tuberculosis* (Mtb), remains a significant global health threat and led to 1.5 million deaths worldwide in 2013 [1]. The only vaccine currently available for TB, *Mycobacterium bovis* bacillus Calmette–Guerin (BCG), is included in neonate or childhood vaccination programs in many countries despite the variable efficacy of protection against pulmonary TB shown in many clinical studies [2, 3]. Nevertheless, recent clinical studies using meta-analyses have shown that the absence of mycobacterial exposure prior to BCG vaccination is associated with higher efficacy [4], and BCG is still advantageous against Mtb infection [5]. Thus, although it is not sufficient to eradicate TB, prime vaccination with BCG is still useful and practical, and prime-boost regimens may provide a more promising vaccine strategy against TB.

BCG is typically administered by the intradermal (i.d.) or subcutaneous (s.c.) route. Considering that TB infections are primarily attained via the airway and because the host immunity to TB is mediated by immune cells in the lungs, many researchers have performed studies to match the route of vaccination to the natural route of infection to obtain higher efficacy. Among these, the most recent study showed that the intranasal (i.n.) administration of BCG showed greater protection compared to s.c. administration, which lasted up to 10 months post-immunization in mice [6]. Additionally, the intratracheal (i.t.) administration of BCG showed better efficacy than s.c. administration, with dose dependency [7]. As BCG-induced immunity would be expected to wane over time [8] and more than 80% of people received this vaccine when they are neonates, boosting the BCG-induced immunity is a more practical strategy than new TB vaccine development.

Recently, many researchers have investigated the potential of various antigens, vectors and adjuvant to boost BCG-induced immunity. Among them, boosting with MVA85A, a modified vaccinia Ankara virus harboring antigen 85A, showed improved protective efficacy in animal models when it was administered intramuscularly, although it did not show protective effects in clinical trials [9]. However, it was demonstrated that aerosol boosting with MVA85A showed enhanced protection and increased immunogenicity compared to intramuscular boosting in animal models [10, 11], although this route of administration has not yet been tested in human clinical trials. Thus, there is substantial evidence that BCG immunization via the airway route, whether used as a single vaccine or a priming vaccine as a heterologous boosting strategy, confers superior protection.

In contrast, there have been only limited studies regarding the protective efficacy of homologous boosting using BCG via the airway route. In addition, revaccination strategies involving the homologous boosting of BCG via the s.c. or i.d. route showed limited protective efficacy in several clinical trials [12–15]. As BCG is a live (albeit attenuated) bacterium, it has the potential to become disseminated and cause disease, especially in patients with primary immunodeficiency [16]; the WHO does not recommend revaccination with BCG for this reason [17]. Further mucosal immunization via the intranasal route has raised safety concerns when subunit vaccines were administered via this route with adjuvants [18, 19]. Considering these factors, we assumed that inactivated BCG would be safer and could be used as an aerosol booster regimen. Nevertheless, one previous study used heat-killed BCG as an antigen in combination with adjuvant as an i.n. booster, but a single boost resulted in no further protection compared with parental immunization in the lungs, despite a significant reduction in the bacterial load in the spleen [20]. Thus, when using BCG for aerosol-based TB vaccine strategies, technical issues such as sustaining the immunogenicity of BCG, the preparation of inactivated BCG, and the number of boosting events must be optimized.

Microbes can be inactivated by several methods, and the preservation of antigenic epitopes is a key factor that affects the efficacy of an inactivated vaccine. The method of inactivation also affects the antigenicity, and thus the efficacy, of a vaccine. For example, numerous studies with the influenza virus (for which most vaccines are derived from chemically inactivated viruses) reported that the antigenic structures might have been altered by chemical treatment, resulting in a failure to induce appropriate immune responses [21–24]. For example, γ-irradiated *Listeria monocytogenes* induced protective T cell responses in mice more efficiently compared to heat-killed bacteria [25]. This higher efficiency is important because the host cellular immunity is important for protection against *L*. *monocytogenes*, similarly to Mtb infection. Thus, we applied a γ-irradiation method to inactivate the live BCG employed as part of a booster regimen. In addition, we hypothesized that an increased number of aerosol boost episodes may enhance the protective efficacy, similar to the dose-dependent protective efficacy observed following i.t. BCG vaccination in mice [7]. To the best of our knowledge, our study is the first to apply a γ-irradiation method for BCG inactivation.

The phenotypic and pathogenic variations among Mtb isolates are important to consider when designing and developing a TB vaccine, and the vaccine should be valid for the most prominent Mtb genotype(s). Because most TB experiments used for vaccine testing have been performed using a laboratory strain (H37Rv), which does not adequately reflect the clinical situation, it is necessary to test the protective efficacy of new vaccines against highly virulent, clinically relevant TB isolates [8]. The W-Beijing *Mycobacterium tuberculosis* family, the predominant genotype of *M*. *tuberculosis* strains, is distributed widely around the world and is highly prevalent in East Asia [26]. An epidemiological study showed that W-Beijing family strains were isolated more frequently from BCG-vaccinated TB patients than from non-immunized patients, suggesting that it may be a selective force for the emergence of the W-Beijing genotype [27]. In addition, a previous study showed that BCG vaccination of mice provided less protection against W-Beijing isolates than Mtb H37Rv [28]. One clinical isolate that caused a TB outbreak, HN878, has been characterized as a hypervirulent strain associated with increased mortality [29], and BCG was shown to have reduced protective efficacy against this strain [30, 31]. Therefore, we tested our hypothesis regarding the boosting strategy by using the clinical hypervirulent strain HN878 in a murine model in this study.

## Materials and Methods

### Ethics statement

All animal experiments were performed in accordance with the Korean Food and Drug Administration (KFDA) guidelines. The experimental protocols used in this study were reviewed and approved by the Ethics Committee and Institutional Animal Care and Use Committee (Permit Number: 2013-0016-03) of the Laboratory Animal Research Center at Yonsei University College of Medicine (Seoul, Korea). Carbon dioxide (CO_2_) was used for euthanasia.

### Mice

Six-week-old female specific pathogen-free C57BL/6 mice were purchased from Japan SLC, Inc. (Shijuoka, Japan). The mice were maintained under barrier conditions in a BL-3 biohazard animal facility at the Yonsei University Medical Research Center with constant temperature (24 ± 1°C) and humidity (50 ± 5%). The animals were fed a sterile commercial mouse diet and provided with water *ad libitum* under standardized light-controlled conditions (12 h light and dark periods). The mice were monitored daily, and none of the mice exhibited any clinical symptoms or illness during this experiment.

### Bacterial strains and preparation of inactivated BCG by γ-irradiation

*M*. *tuberculosis* HN878 was obtained from the strain collections of the International Tuberculosis Research Center (ITRC, Changwon, Gyeongsangnam-do, South Korea). *Mycobacterium bovis* BCG (Pasteur strain 1173P2) was kindly provided by Dr. Brosch at the Pasteur Institute (Paris, France). All mycobacteria used in this study were prepared as described previously [30].

BCG was irradiated using a Gammacell 3000 Elan irradiator (MDS Nordion, Ottawa, Ontario, Canada) with central 35,724 Gy and a minimum of 26,864 Gy for 1.5 h. After irradiation, the bacilli were plated and cultured for six weeks, and the BCG were confirmed to show no growth during this culture.

### Vaccination protocols and experimental infection

The vaccination groups and schedules are shown in Table 1. For priming vaccination, mice were vaccinated with 3 × 10^5^ CFU of BCG via the s.c. route. All aerosol immunizations or TB infections by the aerosol route were performed with a Glas-Col aerosol apparatus (Terre Haute, IN), which is a whole body exposure chamber. For the aerosol immunization of live BCG, the nebulizer compartment was filled with 6 ml of a suspension containing 2 × 10^9^ CFU of BCG. This amount corresponded to each mouse receiving approximately 2 × 10^4^ CFU of BCG. The same amount of BCG (2 × 10^9^ CFU / 6ml) was irradiated and loaded to the nebulizer compartment for aerosol immunization. Six weeks after the final immunization, 5 mice per group were euthanized to analyze the immune responses, and the remaining mice received 200 CFU of HN878 by the aerosol route at 7 weeks after the final immunization.

**Table 1:** Experimental groups in this study.

Group (Animal number)	Priming agent (route)	Boosting agent (route)	Description (Weeks after prime vaccination)
G1 (n = 25)	None	None	Naïve
G2 (n = 25)	None	None	Infection control
G3 (n = 25)	Live BCG (s.c.)	None	Parenteral immunization
G4 (n = 25)	Live BCG (s.c.)	Irradiated BCG (aerosol)	Boosted once (9 w)
G5 (n = 25)	Live BCG (s.c.)	Irradiated BCG (aerosol)	Boosted 3 times (6 w, 9 w and 12 w)
G6 (n = 25)	Live BCG (s.c.)	Live BCG (aerosol)	Boosted once (9 w)
G7 (n = 25)	None	Irradiated BCG (aerosol)	Administered 3 times (6 w, 9 w and 12 w)

### Bacterial counts and histopathological analysis

At 5 and 10 weeks after the HN878 challenge, five mice per group were euthanized with CO_2_, and the lungs and spleens were homogenized. The number of viable bacteria was determined by plating serial dilutions of the organ (left lung or half spleen) homogenates onto Middlebrook 7H11 agar (Difco Laboratories, Detroit, MI) supplemented with 10% OADC (Difco Laboratories), amphotericin B (Sigma-Aldrich, St. Louis, MO) and 2 μg/ml 2-thiophenecarboxylic acid hydrazide (Sigma-Aldrich). Colonies were counted after 2–3 weeks of incubation at 37°C. For the histopathological analysis, the superior lobes of the right lung were stained with hematoxylin and eosin and assessed for the severity of inflammation. The level of inflammation in the lungs was evaluated using the ImageJ (National Institutes of Health, Bethesda, ML) software program, as described previously [32]. In addition, the inflammatory responses were assessed based on lesion size and constitution of immune cells.

### Analysis of memory T cells by flow cytometry

For single-cell preparations, whole spleens and lungs were aseptically removed from 5 mice per group at each euthanized time-point and incubated with RPMI 1640 digestion media (10% fetal bovine serum, 0.1% collagenase type II [Worthington Biochemical Corporation, NJ], 1 mM MgCl_2_, 1 mM CaCl_2_) at 37°C for 30 min. Single-cell suspensions were filtered through a 40 μm cell nylon mesh cell strainer (BD Biosciences, San Diego, CA), treated with red blood cell (RBC) lysing buffer (Sigma-Aldrich, St. Louis, MO) for 5 min, and washed twice with RPMI 1640 medium (BioWest, Nuaillé, France) supplemented with 2% FBS. Single cells were first blocked with Fc Block (anti-CD16/32) for 15 min at 4°C and then stained with BV421-conjugated anti-CD3, PerCp-Cy5.5-conjugated anti-CD4, APC-Cy7-conjugated anti-CD8, PE-conjugated anti-CD44, FITC-conjugated anti-CD62L and APC-conjugated anti-CD127 antibodies for 30 min at 4°C.

### Intracellular cytokine staining

Purified protein derivative (PPD) was kindly provided by Dr. Brennan at Aeras (Rockville, MD), and Ag85A-specific CD4 (Ag85A 261-279aa [THSWEYWGAQLNAMKPDLQ]) and CD8 (Ag85A 2-11aa [SRPGLPVEYL]) T cell peptides were synthesized by AbFrontier company (Young In Frontier, Seoul, Korea). For intracellular cytokine staining, single cell suspensions from immunized animals (2 × 10^6^ cells) were stimulated with PPD (2 μg/ml), Ag85A-specific CD4 or CD8 T cell peptides (2 μg/ml) for 12 hours at 37°C in the presence of GolgiStop (BD Biosciences). Only PPD was used as a stimulant for intracellular cytokine staining following HN878 challenge. Cells were first blocked with Fc Block (anti-CD16/32) for 15 min at 4°C and then stained with BV421-conjugated anti-CD3, PerCp-Cy5.5-conjugated anti-CD4, APC-Cy7-conjugated anti-CD8 and FITC-conjugated anti-CD62L antibodies for 30 min at 4°C. These cells were fixed and permeabilized with a Cytofix/Cytoperm kit (BD Biosciences) according to the manufacturer’s instructions. Intracellular TNF-α, IL-2 and IFN-γ were detected using APC-conjugated anti-TNF-α, PE-Cy7-conjugated anti-IL-2 and PE-conjugated anti-IFN-γ antibodies in a permeation buffer. All antibodies were purchased from eBioscience (San Diego, CA) unless otherwise stated. The cells were analyzed by a FACSverse flow cytometer using the commercially available software program FlowJo (Treestar, Inc., San Carlos, CA).

### Cytokine measurement in culture supernatant

Single cells prepared from the lungs of the immunized or infected mice were stimulated with PPD (2 μg/ml) or Ag85A-specific CD4 or CD8 T cell peptides (2 μg/ml) for 24 h at 37°C. The IFN-γ cytokine levels in the culture supernatant were measured using a commercial ELISA kit (eBioscience) according to the manufacturer’s protocol.

### Antibody titers in serum

The PPD-specific IgG1, IgG2a, IgG2b, IgG2c and total IgG titers in serum were measured as an indicator of the antigen-specific type 1 or type 2 immune responses. Briefly, plates were coated with 1 μg/ml PPD, and HRP-conjugated antibodies against each IgG isotype were used as secondary antibodies. After stopping the reaction, the plates were read within 30 min at 495 nm with a microplate ELISA reader with a cut-off OD of 0.1, obtained using serum from non-immunized and non-infected mice.

### Statistical analysis

The results are reported as the median ± interquartile range (IQR) or mean ± standard deviation (SD). The levels of significance for comparisons between samples were determined by a one-way analysis of variance (ANOVA), followed by Dunnett’s post-test or a two-tailed unpaired *t*-test using the statistical software program GraphPad Prism V5.0 (GraphPad Software, San Diego, CA).

## Results

### Immune responses induced by aerosolized boosting of γ-irradiated BCG in the lungs

**Fig 1:**
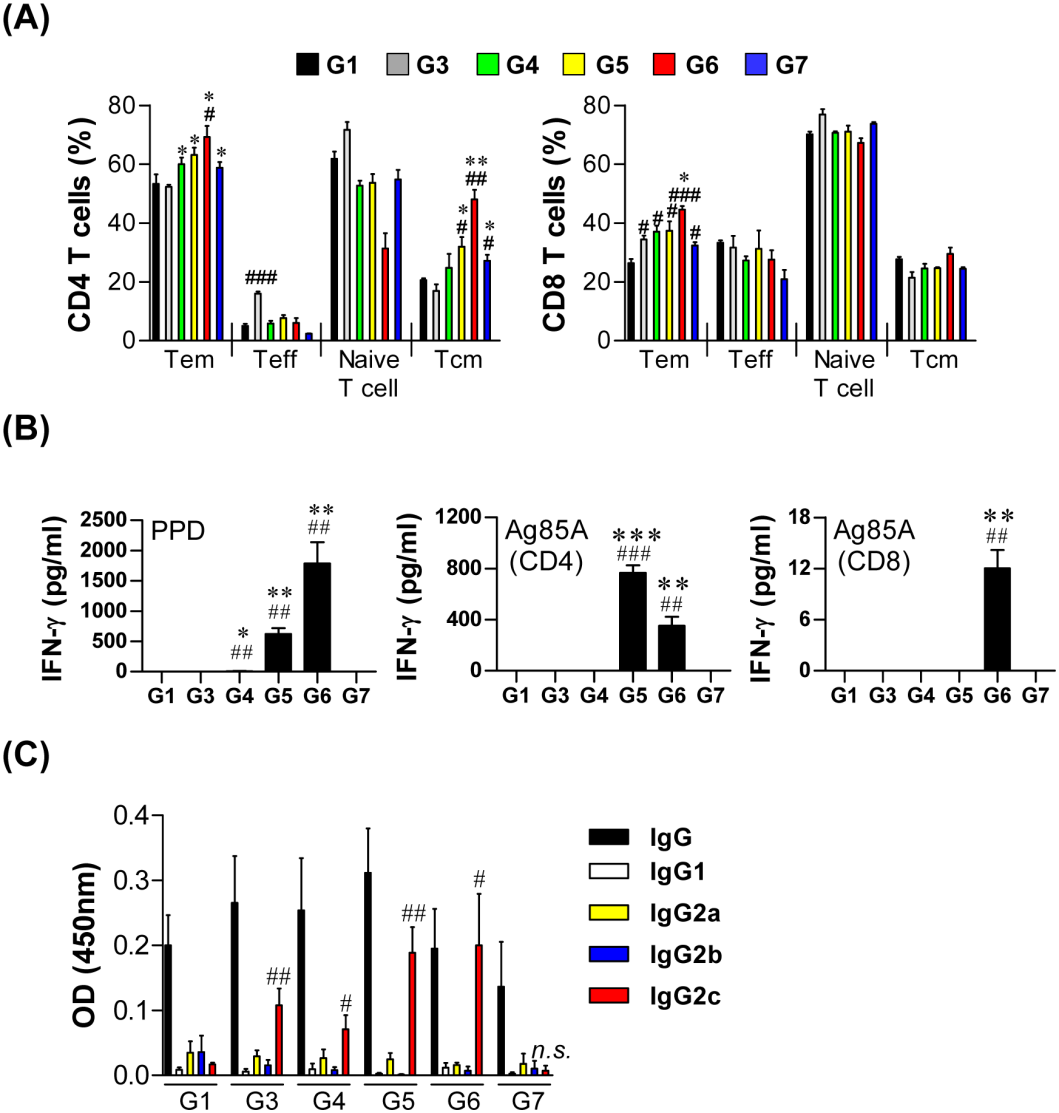
Immunogenicity in the lungs of immunized mice. Three weeks after the final immunization, mice from each group (n = 5) were sacrificed and lung cells were prepared as described in the materials and methods section. The percentages of CD4^+^, CD8^+^ central memory (CD44^hi^CD62L^+^CD127^+^), effector memory (CD44^hi^CD62L^-^CD127^+^), effector (CD44^hi^CD62L^-^CD127^-^), and naïve (CD44^lo^CD62L^+^CD127^+^) T cells were analyzed by flow cytometry (A). A total of 2 × 10^6^ cells were added to each well of microtiter plates and incubated with PPD (2 μg/ml) or Ag85A-specific CD4 or CD8 T-cell peptides (2 μg/ml) for 24 h at 37°C. The IFN-γ concentrations in the suspension were detected using commercial ELISA kits (B). The induction of PPD-specific IgG2c antibodies in the serum from each group of mice (C). The data are presented as the means ± SD from five mice in each group. An unpaired *t*-test was used to determine the significance of differences. A value of *p*<0.05 was considered to be statistically significant. ^#^ *p*<0.05, ^##^ *p*<0.01, and ^###^ *p*<0.001 compared to G1. * *p*<0.05, ** *p*<0.01, and *** *p*<0.001 compared to G3. *n*.*s*.: not significant.

To determine the phenotypic properties of the memory T cells in the lung generated by each boosting strategy, we analyzed the surface expression of CD44, CD62L and CD127 on CD3^+^-gated CD4^+^ and CD8^+^ T cells using flow cytometry (Fig 1A and S1 Fig). For CD4^+^ T cells, the effector memory T cell (T_EM_) population was significantly increased in G6 compared to G1, and all other groups showed a significantly increased T_EM_ population compared to G3 (*p*<0.05). The CD4^+^ central memory T cell (T_CM_) population was significantly increased in G5, G6 and G7 compared to both G1 and G3 (*p*<0.05). Interestingly, BCG immunization did not induce CD4^+^ T_EM_ and T_CM_ populations but did induce the effector T cell population (T_EFF_). For CD8^+^ T cells, all groups showed a significantly increased T_EM_ population compared to G1. In particular, the T_EM_ population in G6 was higher than that in G3 (*p*<0.05), but no other changes were observed in the other population of CD8^+^ cells.

The lung cells were stimulated with PPD or Ag85A-specific CD4 or CD8 T-cell peptides, and the level of IFN-γ was measured to analyze the IFN-γ responses of each boosting strategy (Fig 1B). When stimulated with PPD, the IFN-γ production of each group showed a similar pattern to the population of CD4^+^ T_EM_ cells. However, when stimulated with Ag85A-specific CD4 T cell peptide, only G5 and G6 produced IFN-γ, and the production level was higher in G5 than G6 (*p*<0.05). Only G6 showed IFN-γ production in response to Ag85A-specific CD8 T cell peptides, which was correlated with the increased CD8^+^ T_EM_ population in this group. A further analysis of the PPD-specific antibody titer showed that the mice in all groups exhibited PPD-specific IgG2c responses, an indicator of a Th1-biased response, except G7, which had no live BCG vaccination by either route (Fig 1C).

**Fig 2:**
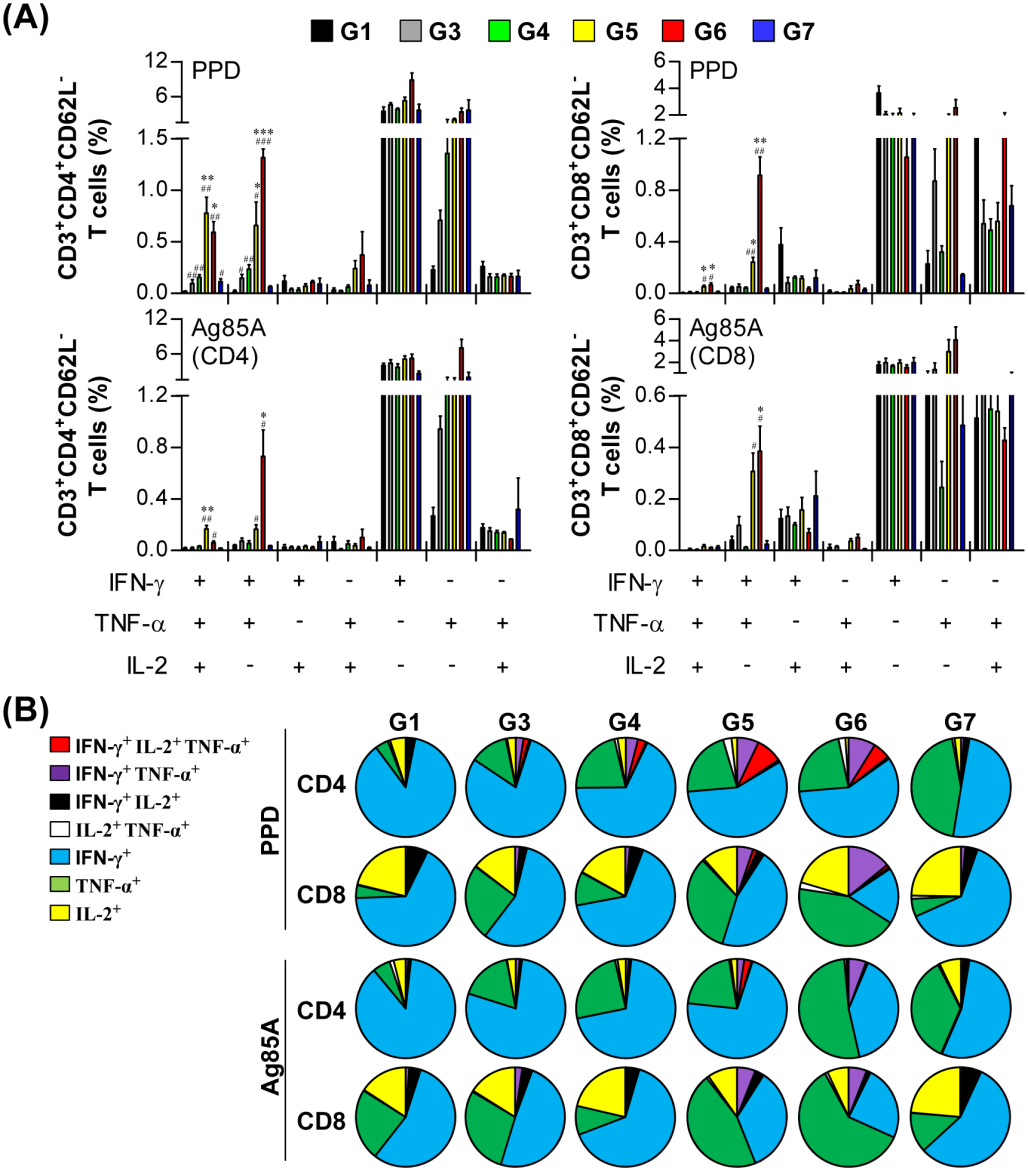
Induction of antigen-specific multifunctional T cells in the lungs of immunized mice. Each group of mice was immunized and sacrificed as described in the materials and methods section. Three weeks after the final immunization, mice from each group (n = 5) were euthanized and their lung cells (2 × 10^6^ cells) were stimulated with PPD (2 μg/ml) or Ag85A-specific CD4 or CD8 T cell peptides (2 μg/ml) for 12 h at 37°C in the presence of GolgiStop. The percentages of antigen-specific CD4^+^CD62L^-^ and CD8^+^CD62L^-^ T cells producing IFN-γ, TNF-α, and/or IL-2 in the cells isolated from the lungs of each group of mice were analyzed by multicolor flow cytometry by gating for CD4^+^ and CD8^+^ lymphocytes (A). Pie charts (B) show the mean frequencies of cells coexpressing IFN-γ, TNF-α, and/or IL-2. The data are presented as the mean ± SD from five mice in each group. An unpaired *t*-test was used to determine the significance of differences. A value of *p*<0.05 was considered to be statistically significant. ^#^ *p*<0.05, ^##^ *p*<0.01, and ^###^ *p*<0.001 compared to G1. * *p*<0.05, ** *p*<0.01, and *** *p*<0.001 compared to G3.

To evaluate the pulmonary immune responses induced by each boosting strategy, we conducted multiparameter flow cytometry with lung cells from immunized mice and measured the percentage of multifunctional T cells that produced IFN-γ plus TNF-α and/or IL-2 (Fig 2 and S1 Fig). In response to PPD stimulation, increased numbers of triple-positive (IFN-γ^+^, TNF-α^+^, and IL-2^+^) and double-positive (IFN-γ^+^ and TNF-α^+^) CD4^+^CD62L^-^ cells were observed in the lungs of immunized mice (*p*<0.05). However, only G5 and G6 showed significantly increased numbers of triple-positive CD4^+^CD62L^-^ cells compared to G3 (*p*<0.05). Interestingly, the percentage of triple-positive and double-positive (IFN-γ^+^ and TNF-α^+^) CD4^+^CD62L^-^ cells did not increase in response to Ag85A stimulation, except in G5 and G6. For the CD8^+^CD62L^-^ cells, both G5 and G6 showed significantly increased numbers of triple-positive and double-positive (IFN-γ^+^ and TNF-α^+^) populations in response to PPD stimulation compared to both naïve cells and G3 cells (*p*<0.05). Moreover, G5 and G6 showed significantly increased Ag85A-specific double-positive (IFN-γ^+^ and TNF-α^+^) CD8^+^CD62L^-^ cells, but only G6 was significantly increased compared to G3 (*p*<0.05). The numbers of multifunctional CD4^+^ and CD8^+^ T cells that produced IFN-γ and IL-2 or TNF-α and IL-2 were not increased by BCG vaccination and/or the additional boosting methods performed in this study. Thus, aerosolized boosting with irradiated BCG three times or with live BCG one time induced an increase in the number of antigen-specific multifunctional CD4^+^ and CD8^+^ T cells in the lungs of mice.

### Protective efficacy of aerosolized boosting in the lungs and spleens of mice after challenge with the *M*. *tuberculosis* HN878 strain

**Fig 3:**
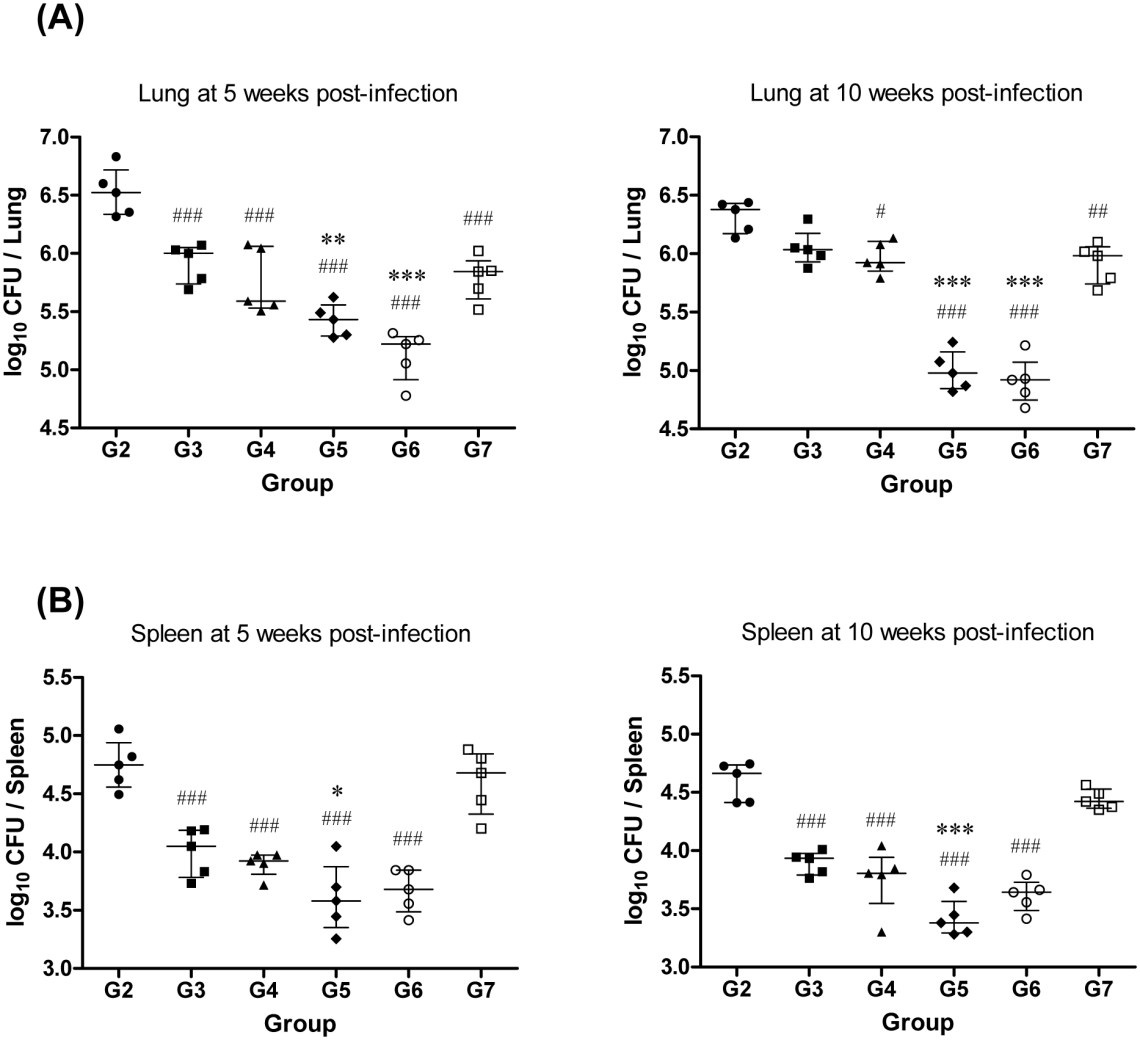
Bacterial loads in the lungs and spleens after immunization and aerosol challenge with the *M*. *tuberculosis* HN878 strain. Seven weeks after the final immunization, mice were challenged with 200 CFU of the *M*. *tuberculosis* HN878 strain via the aerosol route. Five and ten weeks after challenge, the CFU in the lungs (A) and spleens (B) of each group were analyzed by culturing lung and spleen homogenates and enumerating the bacteria. The data are presented as the median ± IQR log_10_CFU/organ (n = 5), and the levels of significance for comparisons between samples were determined by a one-way ANOVA, followed by Dunnett’s test. A value of *p*<0.05 was considered to be statistically significant. ^#^ *p*<0.05, ^##^ *p*<0.01, and ^###^ *p*<0.001 compared to G2. * *p*<0.05, ** *p*<0.01, and *** *p*<0.001 compared to G3.

To compare the protective efficacy, the bacterial burdens in the lungs and spleens of each group of mice were analyzed at 5 and 10 weeks after the challenge (Fig 3). At 5 weeks post-challenge, the bacterial burden in the lungs of mice immunized with BCG via the parenteral route showed less than a 1-log_10_ reduction following the HN878 challenge, although this was a significant difference compared to G2 (*p*<0.001). The other entire group also showed significant bacterial reduction in the lungs compared to G2 (*p*<0.001), and G5 and G6 showed significantly reduced bacterial loads in the lungs of mice, even compared to G3 (*p*<0.01). However, at 10 weeks post-challenge, the bacterial reduction in the lungs of mice immunized with BCG via the parenteral route was diminished. Whereas G4 and G7 showed similar reductions in the bacterial loads in the lungs that were only statistically significant compared to G2, G5 and G6 maintained the bacterial reduction in the lungs, even compared to G3 (*p*<0.001). The bacterial burden in the spleen also showed a similar pattern to the CFU in the lungs, except for G7, which showed no protective efficacy throughout the experiment. Significant bacterial reduction was observed in the spleens of the other entire group at both time points, including the mice immunized with BCG via the parenteral route, which showed no significant bacterial reduction in the lungs at 10 weeks post-challenge. In the spleens, only G5 showed a significant reduction compared to G3. Overall, groups 5 and 6 showed improved protective efficacy against the HN878 challenge in terms of the bacterial reduction in the lungs.

### Histopathological examination of lung tissues after challenge with the *M*. *tuberculosis* HN878 strain

**Fig 4:**
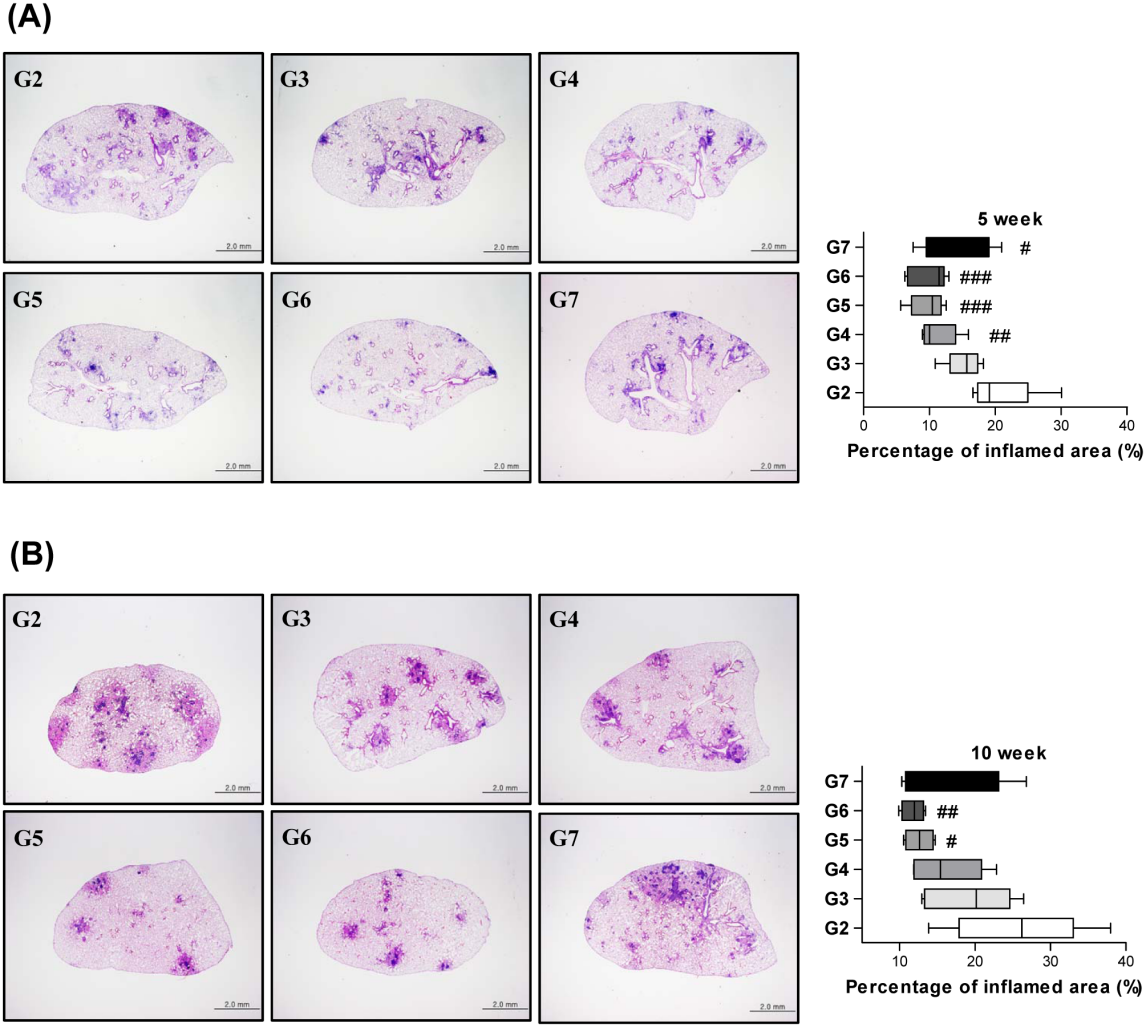
Histopathological lesions of the lungs. Mice were infected with 200 CFU of the *M*. *tuberculosis* HN878 strain via the aerosol route, and the lungs were removed at 5 weeks (A) and 10 weeks (B) post-infection. Representative lung pathological changes from the different groups and the percentages of inflamed areas in lung section are also shown. The data regarding the percentages of the inflamed areas are presented as Whisker box plots (Whiskers represent minimum and maximum values) (n = 5), and a one-way ANOVA followed by Dunnett’s test was used to determine the significance of the findings. A value of *p*<0.05 was considered to be statistically significant. ^#^ *p*<0.05, ^##^ *p*<0.01 and ^###^ *p*<0.001 compared to G2. * *p*<0.05, ** *p*<0.01, and *** *p*<0.001 compared to G3.

To assess the effectiveness of each boosting strategy with regard to the lung pathology, lung slices from each mouse were stained with H&E and the area of inflammation relative to the total lung area was analyzed (Fig 4). At 5 weeks post-infection, G4, G5, G6 and G7 showed significantly reduced inflammation compared to G2 (*p*<0.05). However, no group showed significantly reduced inflammation compared to G3. The pathology of the lungs was considerably more deteriorated at 10 weeks post-infection than at 5 weeks post-infection. At 10 weeks post-infection, only G5 and G6 showed significantly reduced inflammation compared to G2 (*p*<0.05). Whereas the group that received BCG vaccination without boosting showed no significant reduction in lung inflammation, both G5 and G6 showed significantly reduced inflammation in the lungs of mice at both time points.

In histopathological observations, G2 showed granulomatous lesions with larger sizes and at higher frequencies than those of any other BCG-treated groups, irrespective of protocols at 5 weeks post-infection. Therefore, the higher amount of inflammation in control animals would also be due to the larger sizes and numbers of lesions. All of the groups treated with BCG had reduced amounts of granulomatous inflammation compared with the control group. Among the BCG-treated animals, G5 and G6 had fully reduced pathology, with only a few smaller lesions, compared to the other groups. More detailed findings at 5 weeks post-infection revealed that the granulomatous lesions were mainly composed of macrophages and lymphocytes in G2, while numerous lymphocytes had infiltrated in the lesions of G5 and G6 (S2 Fig). Lymphocytic infiltrates present in the lungs of G5 and G6 were often associated with small bronchioles or blood vessels, suggesting an active process of antigen-induced protection related to the T cell-dependent immunity. At 10 weeks post-infection, animals developed progressive pathology, with more extensive inflammatory lesions compared to 5 weeks post-infection. These areas showed abundant vacuolated foamy macrophages in the alveolar lumen admixed with lymphocytes and exudates. The interstitium was wide due to abundant inflammatory infiltrates and fibrosis (S3 Fig). Similar to 5 weeks post-infection, the BCG-boosted animals, particularly G5 and G6, demonstrated smaller and fewer granulomatous pneumonic lesions than G2. Taken together, these pathology data support the stronger protective efficacy of repetitive boosting with irradiated BCG in mice.

### Immunological changes in the lungs of mice after the *M*. *tuberculosis* HN878 challenge

**Fig 5:**
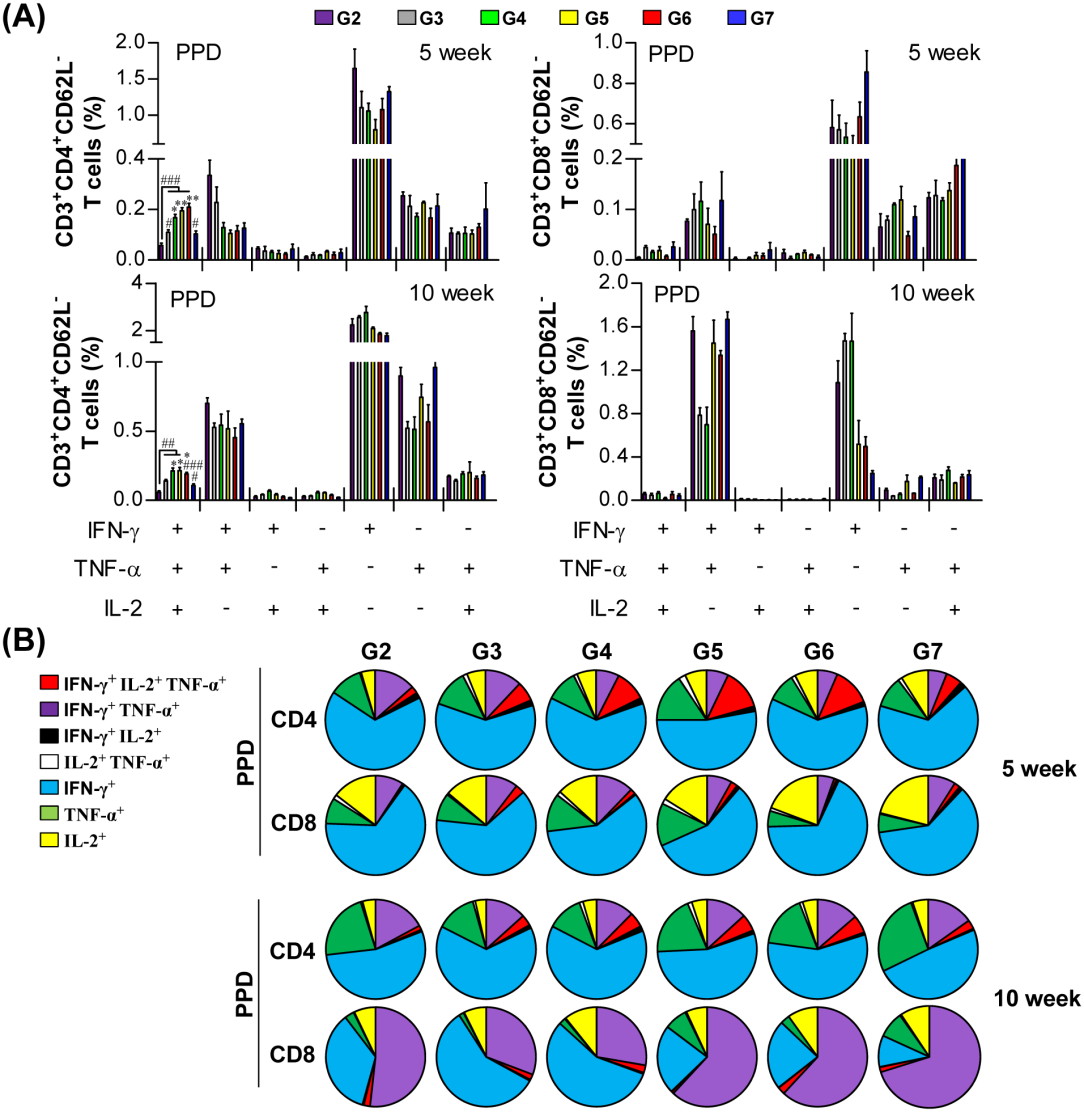
Induction of multifunctional T cells in the lungs of mice after challenge with the *M*. *tuberculosis* HN878 strain. Five and ten weeks post-infection, mice from each group (n = 5) were euthanized and their lung cells (2 × 10^6^ cells) were stimulated with PPD (2 μg/ml) for 12 h at 37°C in the presence of GolgiStop. The percentage of antigen-specific CD4^+^CD62L^-^ and CD8^+^CD62L^-^ T cells producing IFN-γ, TNF-α, and/or IL-2 in the cells isolated from the lungs of each group of mice were analyzed by multicolor flow cytometry by gating for CD4^+^ and CD8^+^ lymphocytes (A). Pie charts (B) show the mean frequencies of cells coexpressing IFN-γ, TNF-α, and/or IL-2. The data are presented as the mean ± SD from five mice in each group. An unpaired *t*-test was used to determine the significance of differences. A value of *p*<0.05 was considered to be statistically significant. ^#^ *p*<0.05, ^##^ *p*<0.01, and ^###^ *p*<0.001 compared to G2. * *p*<0.05, ** *p*<0.01, and *** *p*<0.001 compared to G3.

To determine how the observed changes in the immune responses induced by each boosting strategy influenced the protection against challenge, we followed the immune responses in the lungs of mice after the *M*. *tuberculosis* HN878 challenge. The frequency of PPD-specific triple-positive multifunctional CD4^+^ T cells in the lungs of mice in G5 and G6 was significantly increased before the challenge, and this difference was maintained at both 5 and 10 weeks post-challenge (Fig 5). However, the frequency of triple-positive multifunctional CD8^+^ T cell was diminished after the challenge.

The overall memory and effector T-cell subsets in the lungs of the mice changed after challenge (S4 Fig). At 5 weeks post-challenge, the overall frequency of CD4^+^ T_EM_ cells was decreased but had slightly recovered at 10 weeks post-challenge, whereas the frequency of CD4^+^ T_EFF_ cells was dramatically increased following *M*. *tuberculosis* infection. All groups showed an increased frequency of CD4^+^ T_EM_ cells compared to G2 (*p*<0.01), and this trend was especially notable for G5, which was significantly increased compared to G3 at both 5 and 10 weeks post-infection. The frequency was increased only at 5 weeks for G6. Interestingly, G4 showed an increased frequency of CD4^+^ T_EM_ cells compared to G3 both pre- and post- challenge but without enhanced protective efficacy. For the CD8^+^ T_EM_ cells, a similar pattern was observed except for G5, G6 and G7. The frequency of CD8^+^ T_EM_ cells of these groups was significantly increased at 5 weeks post-infection (*p*<0.05). Surprisingly, the frequency of CD8^+^ T_EFF_ cells was considerably decreased at this time point for G5 and G6. At 10 weeks post-infection, all groups showed an increased CD8^+^ T_EM_ and decreased CD8^+^ T_EFF_ cell frequency compared to the pre-challenge frequencies.

The IFN-γ level was also measured in response to PPD stimulation from the lung cells of each group after challenge (S4 Fig). The IFN-γ production was significantly increased in all groups compared to G2 (*p*<0.05), except for G3 at 10 weeks post-challenge. A similar pattern was observed for the PPD-specific IgG2c responses, with only G5 and G6 maintaining higher levels throughout the experiment (S4 Fig).

## Discussion

The vaccine platforms for TB that have recently been developed vary in their antigens and delivery systems, but most have been designed for parental administration. However, numerous lines of evidence suggest that strengthening the lung immunity (to match the primary route of infection) showed superior protection against TB [6, 7, 10, 11, 20, 33]. Furthermore, it was suggested that aerosol delivery of a TB vaccine directly to the respiratory mucosa might offer physiological and immunological benefits [34]. Although these potential benefits of aerosol TB vaccine strategies have recently been emphasized, there is still a lack of technical and practical means of applying aerosol delivery-based TB vaccines [35].

In this study, we applied a γ-irradiation method to inactivate the live BCG employed as part of an aerosolized booster regimen. As previously reported [31], the protective efficacy of BCG against the hypervirulent HN878 strain is abrogated over time (0.61 log_10_ reduction at 5 weeks vs. 0.27 log_10_ reduction at 10 weeks post-infection). It is still unclear whether this bacterial reduction is due to a complete loss of the BCG-induced immunity or due to a natural reduction of bacteria, which generally occurs at four weeks post-infection in Mtb-resistant mice, such as C57BL/6 mice, due to the host immune response [30]. Nevertheless, our study clearly showed that the protective efficacy in terms of bacterial burden against this Mtb strain was significantly enhanced when irradiated BCG was used to aerogenically boost the response three times. In addition, histopathology data support the enhanced protective efficacy of this boosting strategy. Although we did not evaluate the histopathology of immunized animals prior to Mtb challenge in this study, no obvious gross pathology was observed after final immunization of the repeated BCG aerosol boosting. Nevertheless, more details on histopathological analysis should be obtained to further address the safety concerns involving this boosting strategy.

The protective efficacy of this method was equal to that of vaccination via the aerogenic boosting with live BCG once, and no enhanced protective efficacy was observed following one or three boosting episodes in the absence of priming BCG immunization. It has been recently documented that long-term BCG persistence in the lung by intranasal delivery of a high dose of live BCG (1 × 10^7^ CFU) may provide protective advantages by recruiting Th1-type T-cells in the airway [36]. Although aerosol immunization or boosting with γ-irradiated BCG did not provide the persistency advantage of live BCG, repeated immunization may confer an effect equal to that of persistent live BCG in terms of generation of a protective T cell response and long-lasting immune simulation in the airway.

Strategies strengthening the local immune response have been widely used in several disease models [37, 38]. For example, systemic sanitization (i.e., intraperitoneal injection) with allergen, followed by continuous or intermittent allergen inhalation has been developed to induce an asthmatic-like reaction in the airways. Although many different sensitization and challenge materials have been used, the basic concept and protocol to enhance the local immune response is fundamentally consistent. Given the similarity of the principles developed in models of asthma and arthritis, the repeated exposure of the lungs to γ-irradiated BCG after systemic sensitization with live BCG apparently altered the functional consequences of immune cells, which contributed to preventing Mtb infection.

Additionally, a lack of protection following vaccination with irradiated-BCG three times without priming with live BCG and the multifunctional T cell responses in the spleen shown here (S5 Fig) suggest that both local and systemic T-cell responses are responsible for the protection against disseminated and pulmonary TB. In addition, the elevated PPD-specific IgG2c responses in the serum of the BCG s.c.-primed group also support that BCG priming is a prerequisite to harnessing the local systemic immunity against TB to maintain and enhance the Th1-biased immunity systemically, whereas no significant induction of IgG2c was observed in the group without BCG s.c. priming (Fig 1c). Furthermore, the diminished Th1-biased immune response after the challenge of the hypervirulent Mtb HN878 (Fig 5 and S4 Fig) could result from regulatory T cells, which are known to expand in response to HN878 infection [31].

Although IFN-γ is necessary to control Mtb infection, recent observations have revealed that the IFN-γ response alone is not a reliable marker of vaccine-induced protection, indicating that the IFN-γ response cannot consistently predict the vaccine efficacy [39, 40]. In addition, a single s.c. BCG immunization was able to induce CD4^+^CD44^hi^CD62L^-^CD127^-^ T_EFF_ cells, along with a low level of multifunctional T cells, prior to the Mtb challenge. However, the level of protection is strongly correlated with a significant increase in both the percentage of memory CD4^+^ T cells (both CD4^+^CD44^hi^CD62L^+^CD127^+^ T_CM_ and CD4^+^CD44^hi^CD62L^-^CD127^+^ T_EM_) in the lungs that are capable of producing IFN-γ and the magnitude of antigen-specific multifunctional CD4^+^ T cells producing IFN-γ^+^TNF-α^+^IL-2^+^ and IFN-γ^+^TNF-α^+^ in the lungs. Memory T cell subsets are divided in to T_EM_ and T_CM_ based on their anatomical locations, surface makers, and functional capacities, which are related to the first and second lines of defense, respectively [41]. Moreover, it has been suggested that these cells would be rapidly transited to each subset depending on the presence of antigen [42]. Interestingly, one recent study reported that effector-to-central-memory T-cell transition is minimal, which may also explain the failure of the BCG vaccine in persistent mycobacterial infections [43]. In this study, by aerosol-boosting the mice with γ-irradiated BCG three times, we could successively induce substantial amounts of both types of these T cells in the lungs, whereas parental BCG vaccination failed to induce any significant amounts of these cells in the lungs prior to the Mtb challenge. In addition, the aerosol boosting using γ-irradiated BCG three times successively induced antigen-specific CD4^+^ T cells that were IFN-γ^+^TNF-α^+^IL-2^+^, which were maintained until at least 10 weeks post-infection. In contrast, all groups (including the parental BCG vaccination group) exhibited high levels of IFN-γ-single producer CD4^+^ T cells (Fig 5).

Recently, the protective role of multifunctional T cells against TB in experimental models and human TB cases has been controversial, where multifunctional T cells are considered to be more prone to be associated with active TB in humans [44, 45]. The full pattern and diversity of multifunctional T cells remains to be elucidated. In addition, most human studies of the association of multifunctional T cells with active disease were performed after the progression of TB. In our study, the BCG s.c.-immunized group also showed more generation of multifunctional CD4^+^ T cells producing IFN-γ^+^TNF-α^+^IL-2^+^ at 5 weeks post-Mtb challenge than the infection-only control group (Fig 5A), indicating the presence of ready-to-be expanded multifunctional memory CD4^+^ T cells and that their rapid expansion following the recognition of Mtb infection is critical for vaccine development. Together, these findings indicate that the CD4^+^ T cell populations that mediate protective immunity against TB are likely not IFN-γ-single producing Th1 cells but the generation of high-quality multifunctional cells before Mtb exposure. Moreover, the maintenance of a large number of multifunctional CD4^+^ T cells producing IFN-γ, TNF-α, and IL-2 after Mtb infection appears to be critical to confer protective immunity against hypervirulent Mtb strains.

Although CD8^+^ T cells are known to play important roles in preventing the reactivation of latent TB and in compensating for the CD4^+^ T cell response against primary infection [46], the role of CD8^+^ T_EM_ cells in the vaccine-induced protection against TB remains unclear. In this study, three boost episodes with γ-irradiated bacteria resulted in a similar pattern of CD4^+^ T cell responses to that of live BCG boosting, as well as in a similar level of protection. However, live BCG-boosted mice showed a higher frequency of CD8^+^ T_EM_ cells, together with a higher Ag85A-specific CD8^+^IFN-γ response, than did the γ-irradiated-BCG boosted group (Fig 1A and 1B). However, the frequency of antigen-specific IFN-γ^+^TNF-α^+^ CD8^+^ T cells was increased more significantly in both of these groups than in any of the other groups (Fig 2). Interestingly, the protective role of IFN-γ-secreting CD8 T cells in humans has been confirmed [47], and IFN-γ^+^TNF-α^+^ CD8^+^ T cells are important for the response to latency antigens, including HspX, as determined in long-term latently infected individuals that did not progress to active TB [48]. In addition, the granulysin produced by CD8 T cells directly killed Mtb in vitro [49]. Thus, the precise roles of CD8 T cell subsets in our boosting strategy should be further determined in animal models rather than in murine models because of the absence of granulysin in murine CD8 T cells.

The main strategy of a mucosal vaccine in TB is controlling the early phase of Mtb growth, as there remains an interval of approximately 12 days from infection to the activation of host T cell responses induced by parental immunization [35, 50]. Considering that the containment of Mtb is mediated by lung-resident memory lymphocytes [51], we believe that our strategy may shorten this interval. Consequently, the irradiated BCG-induced protective immunity was more likely dependent on CD4^+^ T cells than CD8^+^ T cells, and an antigen-specific CD8^+^ T-cell response may be required for the optimal protection induced by vaccines. Finally, with regard to the CFU data, the histopathology and immune responses in this study showed that the number of boosting episodes was dependent on the protective immunity and phenotype of T cells. Considering that a single dose of parental BCG vaccine is unlikely to induce mucosal immunity in the airway, it might be one of the reasons that BCG itself is less protective against pulmonary infection causing TB transmission [8]. In this regard, our strategy would potentially contribute to the reduction of TB transmission by increasing the effectiveness against pulmonary TB. The present study has several limitations. First, further studies to optimize the boosting dose and number to provide the greatest activity that does not affect the host safety would help to improve the efficacy of this vaccination strategy. Second, aerosolized boosting strategy employed in this study should be further investigated based on solely on pulmonary delivery for the clinically relevant conclusions, because the Glas-Col aerosol apparatus is a whole body exposure chamber which could influence the gastro-intestinal mucosa as well as the entire respiratory mucosa. Third, the strategy replacing live BCG s.c. priming with γ-irradiated BCG should be further evaluated to ensure safety for immunocompromised populations.

In summary, our present results showed that aerosolized boosting of γ-irradiated BCG is able to elicit Th1-biased immune responses, including antigen-specific memory CD4^+^ T cells concomitantly producing IFN-γ, TNF-α, and IL-2, as well as the IFN-γ response, via both the local and systemic immune systems and to eventually confer improved protection against hypervirulent Mtb HN878 infection following BCG s.c. priming and boosting. Further studies focusing on the long-term protective efficacy and the safety of the dosing of this vaccination regimen are currently underway. The results of these studies and the present results may lead to the development of safer and more effective vaccination strategies against tuberculosis when BCG is used as a priming agent.

## Supporting Information

S1 Fig Gating strategy for flow cytometry analysis. For the memory T-cell analysis, CD4^+^ and CD8^+^ cells were further gated for central memory (CD62L^+^CD127^+^CD44^+^), effector memory (CD62L^-^CD127^+^CD44^+^), effector (CD62L^-^CD127^-^CD44^+^), and naive cells (CD62L^+^CD127^+^CD44^-^) within the CD4^+^ and CD8^+^ T cell populations (A). For the multifunctional T cell analysis, antigen-stimulated lung or spleen cells were identified by intracellular cytokine (IFN-γ, TNF-α, and IL-2) staining based on the CD3 and CD4 or CD8 expression and were further gated on CD62L^lo^ cells (B). The data were collected on a FACSverse flow cytometer, with subsequent analysis using the FlowJo software.
(TIF)

S2 Fig Higher magnification (× 10) of histopathological lesions in the lungs at 5 weeks post-infection. Mice were infected with 200 CFU of the *M*. *tuberculosis* HN878 strain via the aerosol route, and the lungs were removed at 5 weeks post-infection. Hematoxylin and eosin stain. Scale bar = 200 μm.
(TIF)

S3 Fig Higher magnification (× 10) of histopathological lesions in the lungs at 10 weeks post-infection. Mice were infected with 200 CFU of the *M*. *tuberculosis* HN878 strain via the aerosol route, and the lungs were removed at 10 weeks post-infection. Hematoxylin and eosin stain. Scale bar = 200 μm.
(TIF)

S4 Fig Immune responses in the lungs of mice after challenge with the *M*. *tuberculosis* HN878 strain. Five and ten weeks post-challenge, mice in each group (n = 5) were sacrificed and lung cells were prepared as described in the materials and methods section. The percentage of CD4^+^, CD8^+^ central memory (CD44^hi^CD62L^+^CD127^+^), effector memory (CD44^hi^CD62L^-^CD127^+^), effector (CD44^hi^CD62L^-^CD127^-^), and naïve (CD44^lo^CD62L^+^CD127^+^) T cells were analyzed by flow cytometry (A). A total of 2 × 10^6^ cells were added to each well of microtiter plates and incubated with PPD (2 μg/ml) for 24 h at 37°C. The IFN-γ concentrations in the suspensions were detected with commercial ELISA kits (B). The induction of PPD-specific IgG2c antibodies in the serum from each group of mice (C). The data are presented as the mean ± SD from five mice in each group. An unpaired *t*-test was used to determine the significance of differences. A value of *p*<0.05 was considered to be statistically significant. ^#^ *p*<0.05, ^##^ *p*<0.01, and ^###^ *p*<0.001 compared to G2. * *p*<0.05, ** *p*<0.01, and *** *p*<0.001 compared to G3. *n*.*s*.: not significant.
(TIF)

S5 Fig Induction of multifunctional T cells in the spleens of mice after challenge with the *M*. *tuberculosis* HN878 strain. Five and ten weeks post-infection, mice in each group (n = 5) were euthanized and their spleen cells (2 × 10^6^ cells) were stimulated with PPD (2 μg/ml) for 12 h at 37°C in the presence of GolgiStop. The percentage of antigen-specific CD4^+^CD62L^-^ and CD8^+^CD62L^-^ T cells producing IFN-γ, TNF-α, and/or IL-2 in the cells isolated from the lungs of each group of mice were analyzed by multicolor flow cytometry by gating for CD4^+^ and CD8^+^ lymphocytes (A). Pie charts (B) show the mean frequencies of cells coexpressing IFN-γ, TNF-α, and/or IL-2. The data are presented as the means ± SD from five mice in each group. An unpaired *t*-test was used to determine the significance of differences. A value of *p*<0.05 was considered to be statistically significant. ^#^ *p*<0.05, ^##^ *p*<0.01, and ^###^ *p*<0.001 compared to G2. * *p*<0.05, ** *p*<0.01, and *** *p*<0.001 compared to G3.
(TIF)

S1 File NC3Rs ARRIVE Guidelines Checklist (PDF)
