# Supplementary figures and images for: Repeated Aerosolized-Boosting with Gamma-Irradiated Mycobacterium bovis BCG Confers Improved Pulmonary Protection against the Hypervirulent Mycobacterium tuberculosis Strain HN878 in Mice
Source: PLoS One. 2015 Oct 28;10(10):e0141577. doi: 10.1371/journal.pone.0141577 (PMC4624807; doi:10.1371/journal.pone.0141577)

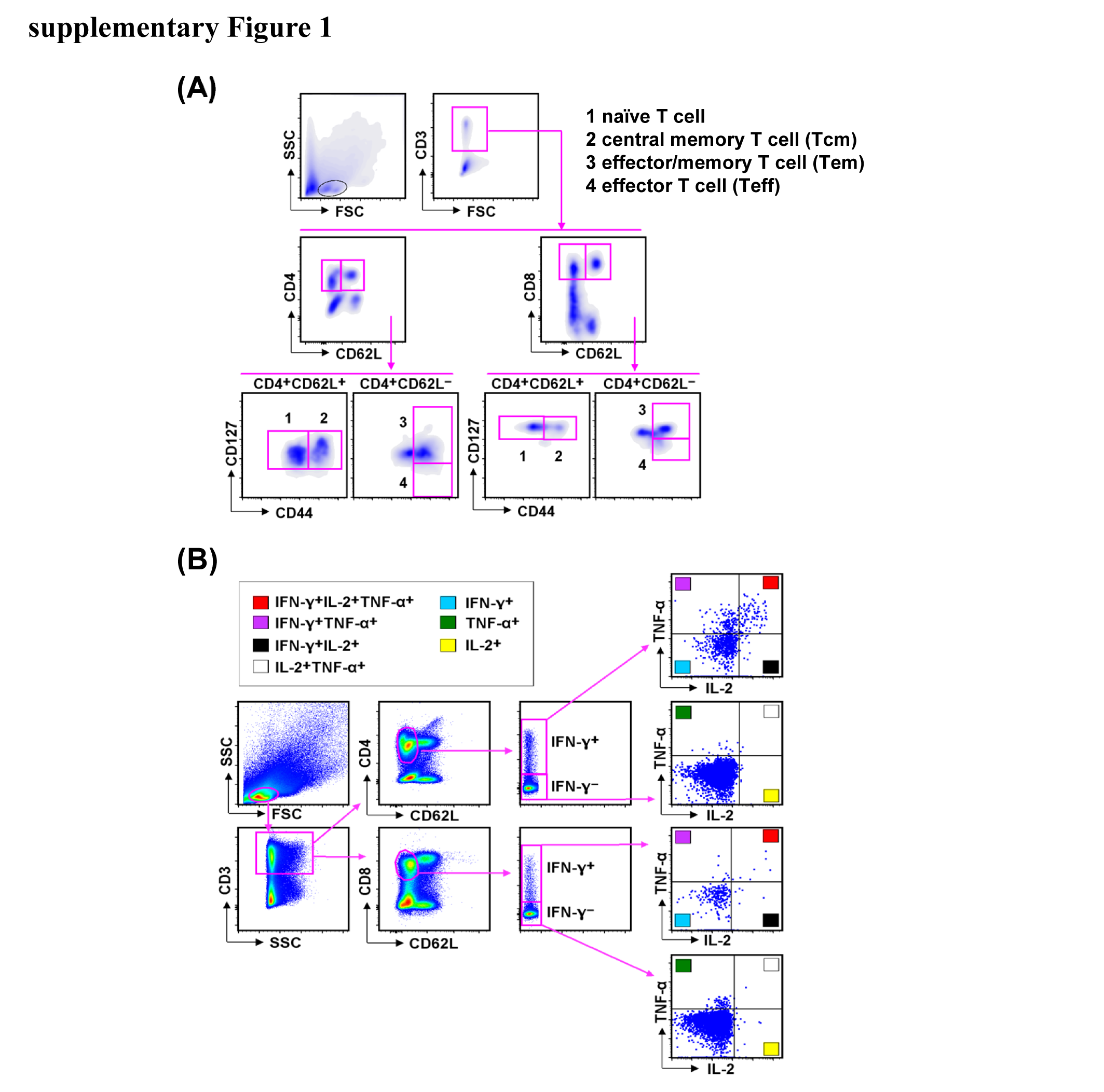

Supplement: S1 Fig — For the memory T-cell analysis, CD4+ and CD8+ cells were further gated for central memory (CD62L+CD127+CD44+), effector memory (CD62L-CD127+CD44+), effector (CD62L-CD127-CD44+), and naive cells (CD62L+CD127+CD44-) within the CD4+ and CD8+ T cell populations (A). For the multifunctional T cell analysis, antigen-stimulated lung or spleen cells were identified by intracellular cytokine (IFN-γ, TNF-α, and IL-2) staining based on the CD3 and CD4 or CD8 expression and were further gated on CD62Llo cells (B). The data were collected on a FACSverse flow cytometer, with subsequent analysis using the FlowJo software. (TIF) [file pone.0141577.s001.tif]

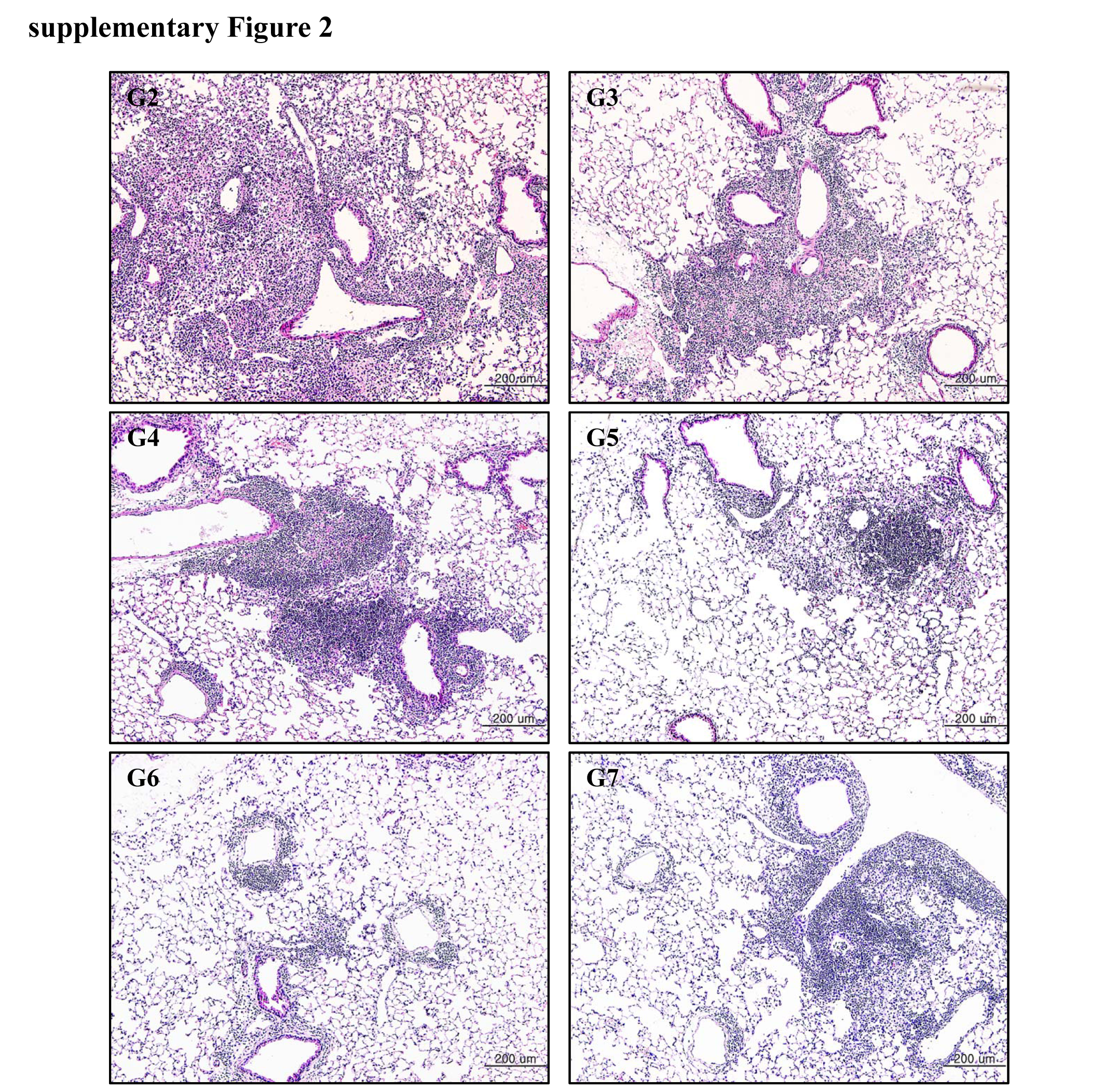

Supplement: S2 Fig — Mice were infected with 200 CFU of the M. tuberculosis HN878 strain via the aerosol route, and the lungs were removed at 5 weeks post-infection. Hematoxylin and eosin stain. Scale bar = 200 μm. (TIF) [file pone.0141577.s002.tif]

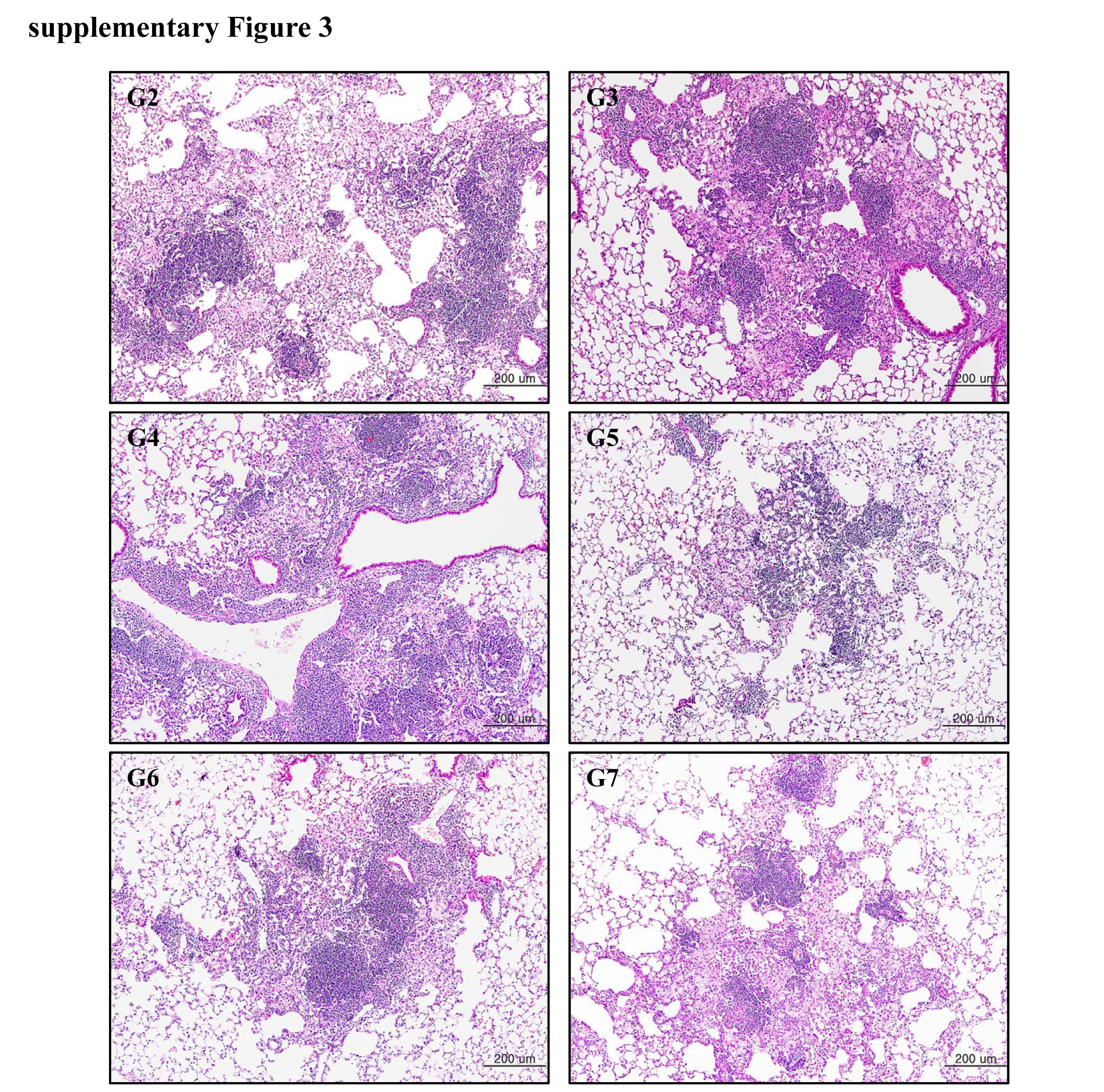

Supplement: S3 Fig — Mice were infected with 200 CFU of the M. tuberculosis HN878 strain via the aerosol route, and the lungs were removed at 10 weeks post-infection. Hematoxylin and eosin stain. Scale bar = 200 μm. (TIF) [file pone.0141577.s003.tif]

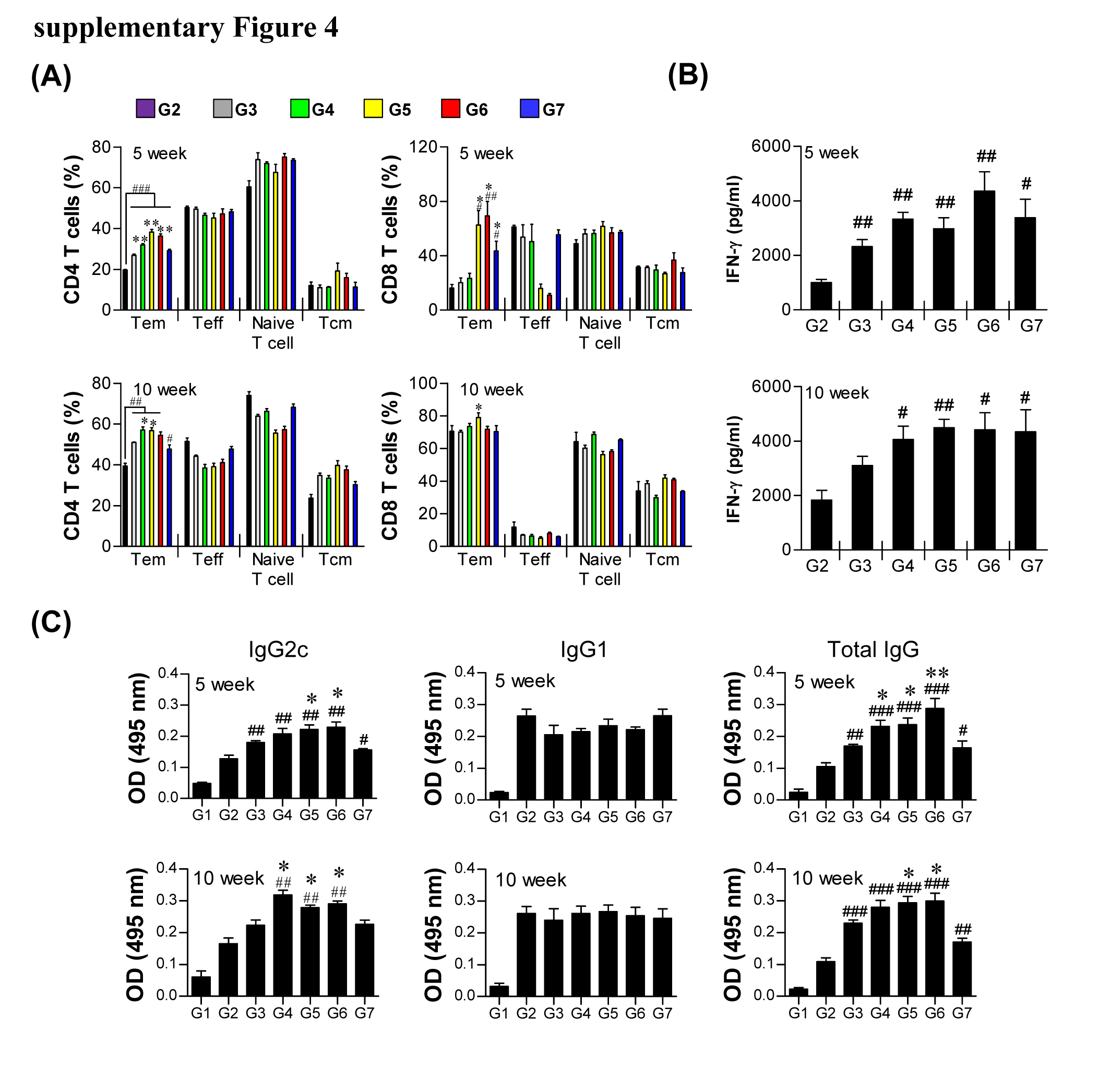

Supplement: S4 Fig — Five and ten weeks post-challenge, mice in each group (n = 5) were sacrificed and lung cells were prepared as described in the materials and methods section. The percentage of CD4+, CD8+ central memory (CD44hiCD62L+CD127+), effector memory (CD44hiCD62L-CD127+), effector (CD44hiCD62L-CD127-), and naïve (CD44loCD62L+CD127+) T cells were analyzed by flow cytometry (A). A total of 2 × 10^6^ cells were added to each well of microtiter plates and incubated with PPD (2 μg/ml) for 24 h at 37°C. The IFN-γ concentrations in the suspensions were detected with commercial ELISA kits (B). The induction of PPD-specific IgG2c antibodies in the serum from each group of mice (C). The data are presented as the mean ± SD from five mice in each group. An unpaired t-test was used to determine the significance of differences. A value of p<0.05 was considered to be statistically significant. # p<0.05, ## p<0.01, and ### p<0.001 compared to G2. * p<0.05, ** p<0.01, and *** p<0.001 compared to G3. n.s.: not significant. (TIF) [file pone.0141577.s004.tif]

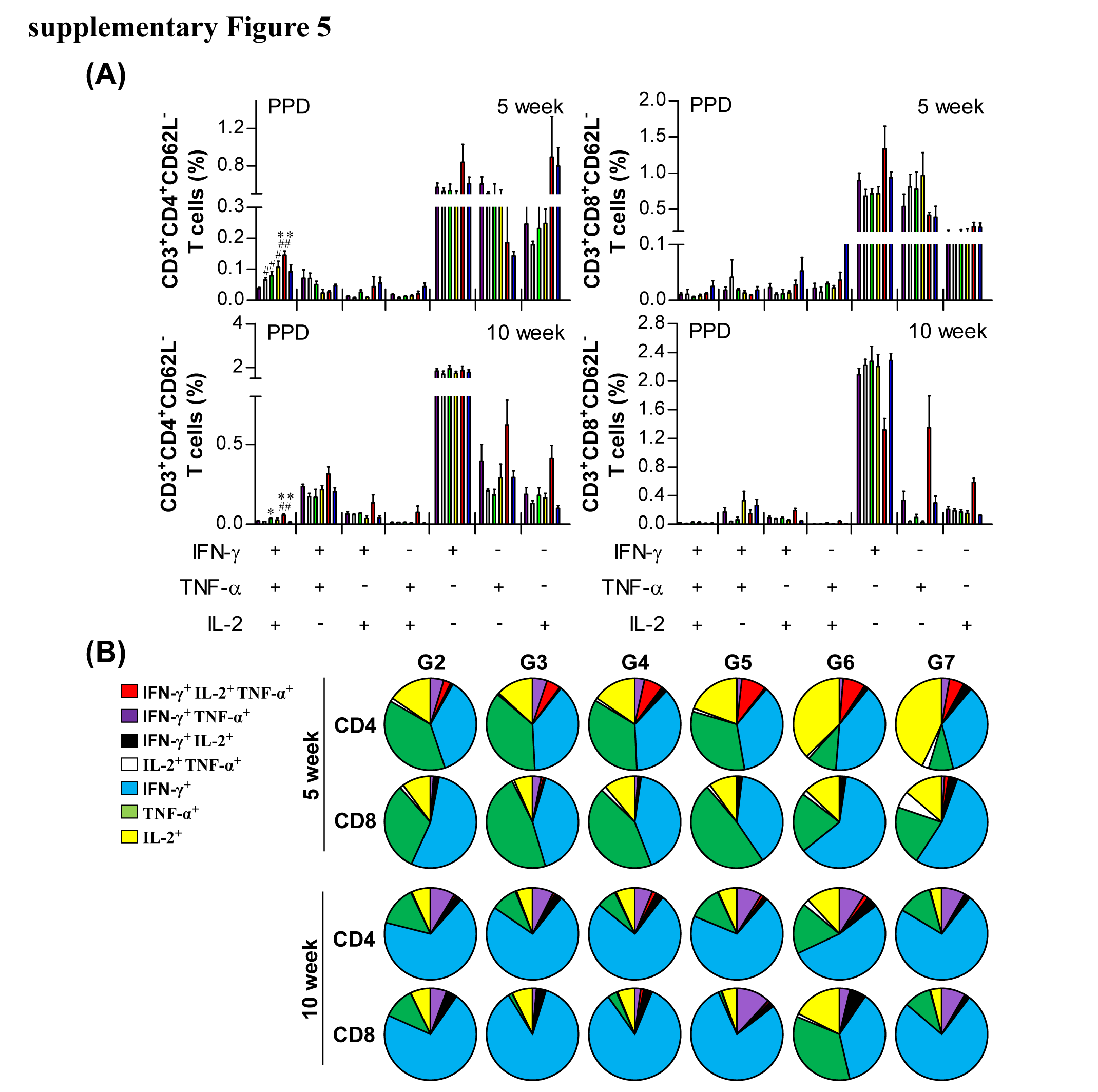

Supplement: S5 Fig — Five and ten weeks post-infection, mice in each group (n = 5) were euthanized and their spleen cells (2 × 10^6^ cells) were stimulated with PPD (2 μg/ml) for 12 h at 37°C in the presence of GolgiStop. The percentage of antigen-specific CD4+CD62L- and CD8+CD62L- T cells producing IFN-γ, TNF-α, and/or IL-2 in the cells isolated from the lungs of each group of mice were analyzed by multicolor flow cytometry by gating for CD4+ and CD8+ lymphocytes (A). Pie charts (B) show the mean frequencies of cells coexpressing IFN-γ, TNF-α, and/or IL-2. The data are presented as the means ± SD from five mice in each group. An unpaired t-test was used to determine the significance of differences. A value of p<0.05 was considered to be statistically significant. # p<0.05, ## p<0.01, and ### p<0.001 compared to G2. * p<0.05, ** p<0.01, and *** p<0.001 compared to G3. (TIF) [file pone.0141577.s005.tif]
